# Ethical, Social & Psychological Challenges of Head Transplantation in Human

**Published:** 2018-09

**Authors:** Dariush D. FARHUD

**Affiliations:** 1. School of Public Health, Tehran University of Medical Sciences, Tehran, Iran; 2. Dept. of Basic Sciences/Ethics, Iranian Academy of Medical Sciences, Tehran, Iran

Many people remember the story of Frankenstein by Mary Shelley published in 1818. It was a story of an ambitious young scientist, linking the pieces of dead bodies and actions related to them created an uncontrollable creature that overcame his creator. Readers found the story only as a myth and imagination of the writer. In twenty-first century, with the development of medical technologies, the world of science will may witness the realization of the dream.

Today, the transplant surgeries have common features (autograft, allograft, isograft, and xenograft). Head transplant is an advance surgery that transplants the head of one organism to another. This kind of surgery was performed successfully on mice, dogs and monkeys (1) (Fig. 1). Sergio Canavaro had claimed that he would go to transplant the first head of human in 2017. He was supposed to transplant the head of a Russian subject with neuromuscular disease to the body of an individual with cerebral death (2).

**Fig. 1:**
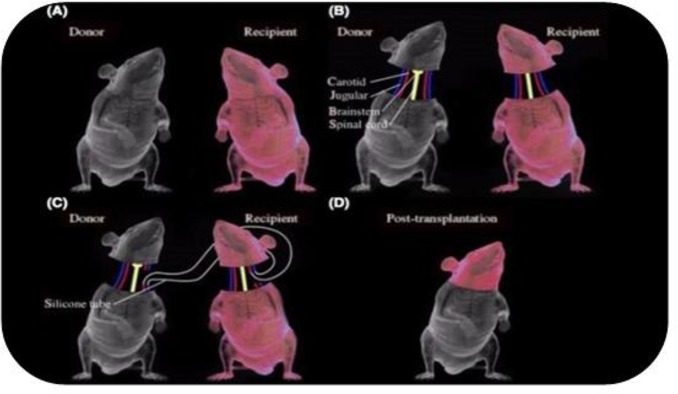
Head transplant surgery on Mouse (3)

Although successful progress of such surgeries is considered wonderful advance in medical sciences and would change the life of human being, it follows the important challenges in public health such as psychological and ethical challenges. Therefore, it is necessary to predict and control these challenges, primarily.

The most important challenge in public health is related to individual identity. Identity is a social and psychological factor and it includes all attitudes, characteristics and feeling of a person that differentiates him/her from others and it comprises the “self” (4). Self-imagination is the important factor that comprises identity. Various evidences advocate the close relationship between formation of identity and body image and it is stated that body identity has a clear role in emergence of cognition and personality (5). Self-imagination has a significant impact on self-esteem and life satisfaction (6).

Since the head is a symbol of individual identity, in head transplant surgeries, the body is seen as a donor organ and the head as a recipient. Hence, dealing with new organ may lead to significant challenges (7), that it changes cognition, skills, character, behavior and personality. Inevitably, they may be confused about their identity.

Since the cognition and personality depends on the sensory/motor experiences that develop during the growth period, the subjects with head transplant may be faced with the new organ strangely (7). As the new organ provides new capabilities, the recipient may experience considerable problems. There are various uncertainties. For instance: the habit of walking, movement of hands, gestures and lateralization. How do these functions? How the new organ reacts to tics and obsessions? Moreover, to which family does the new combination belong?

Totally, it can be said that any change in body identity is the greatest challenge for individual with head transplant. According to the viewpoint of some experts, head transplant with potential effects on identity leads to the psychological disorders like mood disorder, psychosis, suicide (8). This perspective results from observation of patients with surgeries like hand, foot, heart, liver transplant. Sex change operations in transsexual persons they present depression and other psychological disorders most commonly.

There are also some ethical challenges in head transplant surgeries that must be considered. From the last decades, ethics was considered in theories, hypothesis, social and religious discussions and today medical ethics is focused on four fundamental principles: Autonomy, beneficence, non-maleficence and justice (9, 10). In head transplant, all of the principles will be considered. The first question is that: Is the beneficence of surgery is more than its maleficence? Is the autonomy of patients respected? And so on (Fig. 2). Generally, the most important ethical challenges in head transplant are as followed:

- Respecting the fundamental principles of ethics (autonomy, beneficence, non-maleficence and justice)
- Ensuring the brain death of the donor body
- Considering the biological differences of receiver and donor
- Transplantation must be based on the success of operation, not the power and wealth of the donor and recipient.
- Considering the feelings of the donator’s family, since, they will be faced with serious challenges about the living body.
- Preventing the possible abuse of these transplants by the powerful and profiteers.

**Fig. 2:**
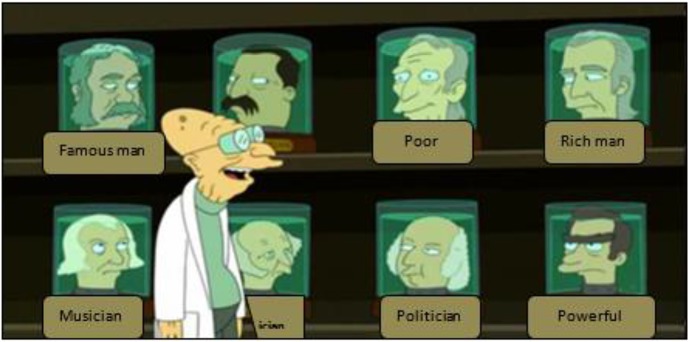
Prioritize the head transplant (11)

## Conclusion

The medical world by being on the verge of realizing a longtime dream adventure (head transplantation surgeries), should be ready for possible side effects. The most common challenges are psychological, social and ethical challenges. In domain of mental health, the increase of psychological disorders that result from individual identity confusion is to be considered. In domain of ethics, head transplant leads to different issues and hence, fundamental principles of medical ethics are challenged. Therefore, the preparation of ethical codes for these advances is absolutely necessary.
